# Membranized Coacervate Microdroplets: from Versatile Protocell Models to Cytomimetic Materials

**DOI:** 10.1021/acs.accounts.2c00696

**Published:** 2023-01-10

**Authors:** Ning Gao, Stephen Mann

**Affiliations:** † Max Planck-Bristol Centre for Minimal Biology, School of Chemistry, University of Bristol, Cantock’s Close, BristolBS8 1TS, United Kingdom; ‡ Centre for Protolife Research and Centre for Organized Matter Chemistry, School of Chemistry, University of Bristol, Cantock’s Close, BristolBS8 1TS, United Kingdom; § School of Materials Science and Engineering, Shanghai Jiao Tong University, 800 Dongchuan Road, Minhang District, Shanghai200240, PR China; ∥ Zhangjiang Institute for Advanced Study (ZIAS), Shanghai Jiao Tong University, 429 Zhangheng Road, Shanghai201203, PR China

## Abstract

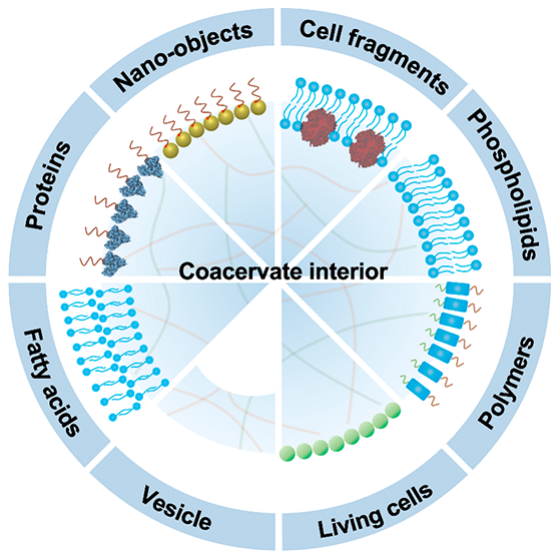

Although complex coacervate microdroplets derived from associative phase separation of counter-charged electrolytes have emerged as a broad platform for the bottom-up construction of membraneless, molecularly crowded protocells, the absence of an enclosing membrane limits the construction of more sophisticated artificial cells and their use as functional cytomimetic materials. To address this problem, we and others have recently developed chemical-based strategies for the membranization of preformed coacervate microdroplets. In this Account, we review our recent work on diverse coacervate systems using a range of membrane building blocks and assembly processes. First, we briefly introduce the unusual nature of the coacervate/water interface, emphasizing the ultralow interfacial tension and broad interfacial width as physiochemical properties that require special attention in the judicious design of membranized coacervate microdroplets. Second, we classify membrane assembly into two different approaches: (i) *interfacial self-assembly* by using diverse surface-active building blocks such as molecular amphiphiles (fatty acids, phospholipids, block copolymers, protein–polymer conjugates) or nano- and microscale objects (liposomes, nanoparticle surfactants, cell fragments, living cells) with appropriate wettability; and (ii) *coacervate droplet-to-vesicle reconfiguration* by employing auxiliary surface reconstruction agents or triggering endogenous transitions (*self-membranization*) under nonstoichiometric (charge mismatched) conditions. We then discuss the key cytomimetic behaviors of membranized coacervate-based model protocells. Customizable permeability is achieved by synergistic effects operating between the molecularly crowded coacervate interior and surrounding membrane. In contrast, metabolic-like endogenous reactivity, diffusive chemical signaling, and collective chemical operations occur specifically in protocell networks comprising diverse populations of membranized coacervate microdroplets. In each case, these cytomimetic behaviors can give rise to functional microscale materials capable of promising cell-like applications. For example, immobilizing spatially segregated enzyme-loaded phospholipid-coated coacervate protocells in concentrically tubular hydrogels delivers prototissue-like bulk materials that generate nitric oxide *in vitro*, enabling platelet deactivation and inhibition of blood clot formation. Alternatively, therapeutic protocells with *in vivo* vasoactivity, high hemocompatibility, and increased blood circulation times are constructed by spontaneous assembly of hemoglobin-containing cell-membrane fragments on the surface of enzyme-loaded coacervate microdroplets. Higher-order properties such as artificial endocytosis are achieved by using nanoparticle-caged coacervate protocell hosts that selectively and actively capture guest nano- and microscale objects by responses to exogenous stimuli or via endogenous enzyme-mediated reactions. Finally, we discuss the current limitations in the design and programming of membranized coacervate microdroplets, which may help to guide future directions in this emerging research area. Taken together, we hope that this Account will inspire new advances in membranized coacervate microdroplets and promote their application in the development of integrated protocell models and functional cytomimetic materials.

## Key References

- GaoN.; XuC.; YinZ.; LiM.; MannS.Triggerable protocell capture in nanoparticle-caged coacervate microdroplets. J. Am. Chem. Soc.2022, 144, 3855–3862, 10.1021/jacs.1c1141435192333PMC9097475.^1^ A nanoparticle-caged coacervate protocell model is developed which enables selective and active capture of guest objects by exogenous stimuli or endogenous enzyme reactions.
- LiuS.; ZhangY.; LiM.; XiongL.; ZhangZ.; YangX.; HeX.; WangK.; LiuJ.; MannS.Enzyme-mediated nitric oxide production in vasoactive erythrocyte membrane-enclosed coacervate protocells. Nat. Chem.2020, 12, 1165–1173. 10.1038/s41557-020-00585-y33219364.^2^ Spontaneous assembly of hemoglobin-containing cell membrane fragments on the surface of enzyme-loaded coacervate microdroplets delivers therapeutic protocells capable of high hemocompatibility, increased blood circulation times, and blood vessel vasodilation.
- TianL.; LiM.; PatilA. J.; DrinkwaterB. W.; MannS.Artificial morphogen-mediated differentiation in synthetic protocells. Nat. Commun.2019, 10, 3321. 10.1038/s41467-019-11316-431346180PMC6658542.^3^ Spatially organized populations of coacervate vesicles with structural and functional diversity are generated by exposing thousands of identical coacervate microdroplets to morphogen gradients, offering an approach to integrating out-of-equilibrium processes into the design of consortia of diverse cell-like entities with graded complexity.
- YinZ.; TianL.; PatilA. J.; LiM.; MannS.Spontaneous membranization in a silk-based coacervate protocell model. Angew. Chem. Int. Ed.2022, 61, e20220230210.1002/anie.202202302PMC930665735176203.^4^ An alginate/silk coacervate protocell was developed which enables enzyme-mediated reversible membranization in the absence of auxiliary complexation agents.

## 1 Introduction

Complex coacervate microdroplets are produced by liquid–liquid phase separation of oppositely charged polyelectrolytes and have diverse applications in areas such as synthetic biology, cytomimetic engineering, and microreactor technology.^5,6^ Compared with conventional membrane-bounded water-filled protocells such as liposomes,^7^ polymersomes,^8^ colloidosomes,^9^ and proteinosomes,^10^ the membrane-less molecularly crowded milieu of coacervate-based protocells resembles the cytoplasmic matrix of living cells.^11^ As the condensed polyelectrolytes comprise approximately 30% of the droplet volume, the inherent chemically enriched interior provides an effective reaction crucible for transformations such as ribonucleic acid catalysis, gene expression, and enzymatic cascades.^12^ Significantly, a range of molecular interactions involving charge complementarity, hydrophobicity, π–π stacking, and hydrogen bonding facilitate the spontaneously uptake and retention of diverse client components from the dilute continuous phase to produce molecularly crowded microenvironments with high localized concentrations of functional components^13^ that support metabolic reactions, chemical communication pathways, and structural integration.^14,15^ Moreover, the dynamic assembly/disassembly and multiple-phase condensation of coacervate microdroplet systems provide unprecedented opportunities for the spatiotemporal control of microscale organization.^16^

Although coacervate microdroplets prepared by polyelectrolyte complexation or single-component liquid–liquid phase separation^17,18^ have been broadly investigated as molecularly crowded model protocells, the absence of an enclosing membrane is a potential drawback for cytomimetic modeling and the development of functional cell-like materials.^19^ In particular, coacervate-based protocells are susceptible to fusion and sensitive to salt/pH-induced instability, which ultimately gives rise to coalescence into a bulk phase over several minutes or hours. Furthermore, although molecules in the continuous phase can be readily sequestered and retained within the coacervate interior, the membraneless system remains open to the external environment such that the nonequilibrium conditions required for advanced cytomimetic modeling are difficult to establish at the coacervate/water interface.

Given these considerations, we and others have recently developed pathways to membranized coacervate microdroplets as a step toward molecularly crowded model protocells with increased stability, controllable semipermeability, multitiered organization and programmable functionality. In this Account, we describe progress in our laboratory over the last five years on the bottom-up construction of diverse membrane-enclosed coacervate droplets and their attendant cytomimetic properties arising from the synergistic integration of functional components. We classify current strategies into two alternative approaches involving (i) *interfacial self-assembly*, in which molecular amphiphiles (fatty acid, phospholipids, block copolymers, protein–polymer conjugates) and amphiphilic nano- and microscale objects (liposomes, Janus nanoparticles, cell fragments, and living cells) are used as surface-active membrane building blocks; and (ii) *endogenous reconfiguration*, in which auxiliary reconstruction chemicals or internalized enzyme networks are employed to induce spontaneous morphological transitions within the coacervate microdroplets that result in membranization. In both cases, membranization results in diverse cytomimetic properties, including selective membrane permeability, microscale reactivity, chemical signaling, and artificial phagocytosis, which provide opportunities for developing new technologies at the synthetic cell/living cell interface.

## 2 Membranized Coacervate Microdroplets via Interfacial Self-Assembly

**Figure 1:**
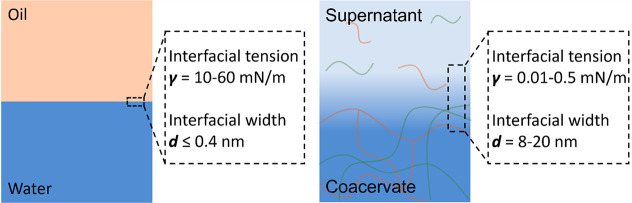
Liquid interfaces for membrane assembly. Scheme showing key properties of water/oil and coacervate/supernatant interfaces. The interfacial width is the distance between two locations where the densities of the coacervate and supernatant are 90% of their own bulk densities.

In principle, a straightforward strategy for achieving coacervate droplet membranization is via the bottom-up self-assembly of molecular amphiphiles as typically used to prepare water droplet-in-oil emulsions. The latter possess a high interfacial tension (10–60 mN/m) and narrow interfacial width (<0.4 nm, Figure 1),^20^ so that molecular amphiphiles such as fatty acids and phospholipids spontaneously self-assemble at the water/oil interface to produce closely packed supramolecular membranes and a concomitant decrease in interfacial tension. In contrast, coacervate droplets and their surrounding continuous phase are water-based, giving rise to a very low interfacial tension, typically between 0.01 and 0.5 mN/m, which is approximately 100–1000 times lower than measured for the oil/water interface (Figure 1).^13,21^ Thus, molecular amphiphiles do not readily assemble at the coacervate/water interface and tend to be accumulated within the coacervate interior due to the decreased dielectric constant.^22^ Moreover, the estimated coacervate/water interfacial width is 8–20 nm,^23^ indicating that conventional molecular surfactants are not large enough to span the coacervate/supernatant interface as a single monolayer. While the structure and composition of the diffuse interfacial layer is not known in detail, the boundary domain is intrinsically dynamic and therefore can be reconfigured under nonstoichiometric (charge-mismatched) conditions to trigger the spontaneous membranization and formation of coacervate vesicles (see section 3).

**Figure 2:**
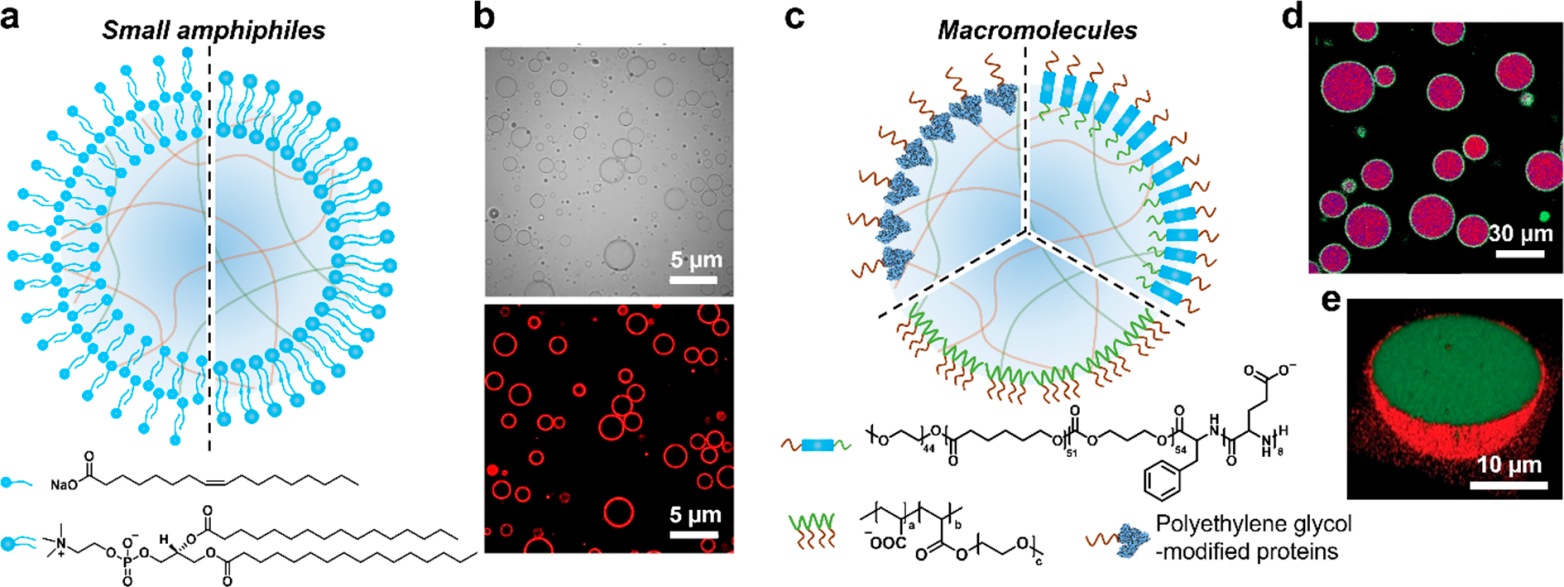
Membranized coacervate microdroplets based on molecular/macromolecular interfacial self-assembly. (a) Scheme showing coacervate microdroplets coated with a multilayer fatty acid (left side)^24^ or a phospholipid bilayer (right side).^27^ (b) Bright-field (top) and fluorescence (bottom) images of phospholipid (DPPC, Dil stain, red fluorescence) bilayer-bounded DEAE-dextran/DNA coacervate microdroplets. Reproduced with permission from ref (27). Copyright 2021 American Chemical Society. (c) Scheme showing coacervate microdroplets coated with macromolecules (terpolymer, top right),^30^ PEG-modified proteins (top left),^32^ or comb polyelectrolytes (bottom).^33^ (d) Confocal laser scanning microscopy (CLSM) image showing terpolymer-bounded coacervate microdroplets (purple). Reproduced with permission from ref (30). Copyright 2017 American Chemical Society. (e) 3D CLSM image showing PEG-modified protein-coated coacervate microdroplet: red fluorescence, RITC-labeled protein; green fluorescence, FITC-tagged coacervate. Reproduced with permission from ref (32). Copyright 2019 Wiley-VCH.

We initially investigated the possibility of coacervate droplet membranization as a plausible protocell model for the emergence of compartmentalization on the early earth.^24,25^ Specifically, we developed a method in which negatively charged fatty acid molecules (sodium oleate) are spontaneously assembled onto the surface of positively charged coacervate microdroplets by electrostatic interfacial interactions.^24^ Assembly of the molecular amphiphile is achieved using oleate concentrations below the critical micelle concentration to minimize competing fatty acid vesicle formation and give rise to stable coacervate microdroplets coated in a semipermeable multilayered membrane (Figure 2a). The methodology is applicable to diverse coacervates prepared from binary mixtures of cationic polyelectrolytes (oligolysine, polylysine, polydiallydimethylammonium chloride (PDDA)) and anionic biomolecules (adenosine triphosphate (ATP), polyribonucleotides (RNA)). A similar strategy has been employed to prepare coacervate droplets coated in a multilayered phospholipid membrane.^26^

Having established the possibility of molecular amphiphile self-organization on the diffuse coacervate/water interface, we extended this approach for the construction of giant coacervate vesicles as an integrated pathway to cytomimetic modeling.^27^ Specifically, we assembled a unilamellar phospholipid bilayer on the surface of preformed diethylaminoethyl-dextran (DEAE-dextran)/DNA coacervate microdroplets using an ethanol solution of the zwitterionic phospholipid 1,2-dipalmitoyl-*sn*-glycero-3-phosphocholine (DPPC). As DPPC has good and poor solubility in ethanol and water, respectively, addition of excess DPPC results in strong interactions at the coacervate microdroplet interface to produce a continuous unilamellar phospholipid bilayer surrounding the coacervate phase (Figure 2a,b). In related studies, we decorated the outer surface of phospholipid-enveloped PDDA/DNA coacervate droplets with an array of lipophilically modified enzymes to produce a stable biochemical reaction platform for the construction of a model prototissue.^28^ Populations of giant coacervate vesicles have also been prepared by hydration of solid phospholipid films in the presence of coacervate droplets.^29^ The method gives rise to coacervate vesicles comprising single- or multilayered shells with continuous or discontinuous membranes depending on the phospholipids and polyelectrolytes used in the assembly system.

As an alternative approach, surface-active macromolecules have been employed to stabilize the diffuse coacervate/water interface, enclosing the coacervate microdroplets in a continuous polymeric membrane (Figure 2c). For example, van Hest and colleagues used a terpolymer comprising polyethylene glycol (PEG), poly(caprolactone-gradient-trimethylene carbonate, PCLgTMC), and poly(glutamic acid, pGlu) to prepare membranized coacervate droplets.^30^ Interfacial assembly of a terpolymer monolayer was attributed to the strong interactions of PEG and pGlu with the continuous water phase and coacervate droplet, respectively, while the PCLgTMC domains provided the required flexibility for dynamic reorganization via hydrophobic chain association. Critically, the terpolymer was ca. 16–18 nm in size, which was sufficient to span the all-water interface (Figure 2c,d).^31^ Similarly, membrane building blocks have been synthesized by grafting PEG onto proteins such as bovine serum albumin, glucose oxidase (GOx), and horseradish peroxidase (HRP) (Figure 2e)^32^ or by using comb polyelectrolytes,^33^ indicating the generality of this membranization strategy.

**Figure 3:**
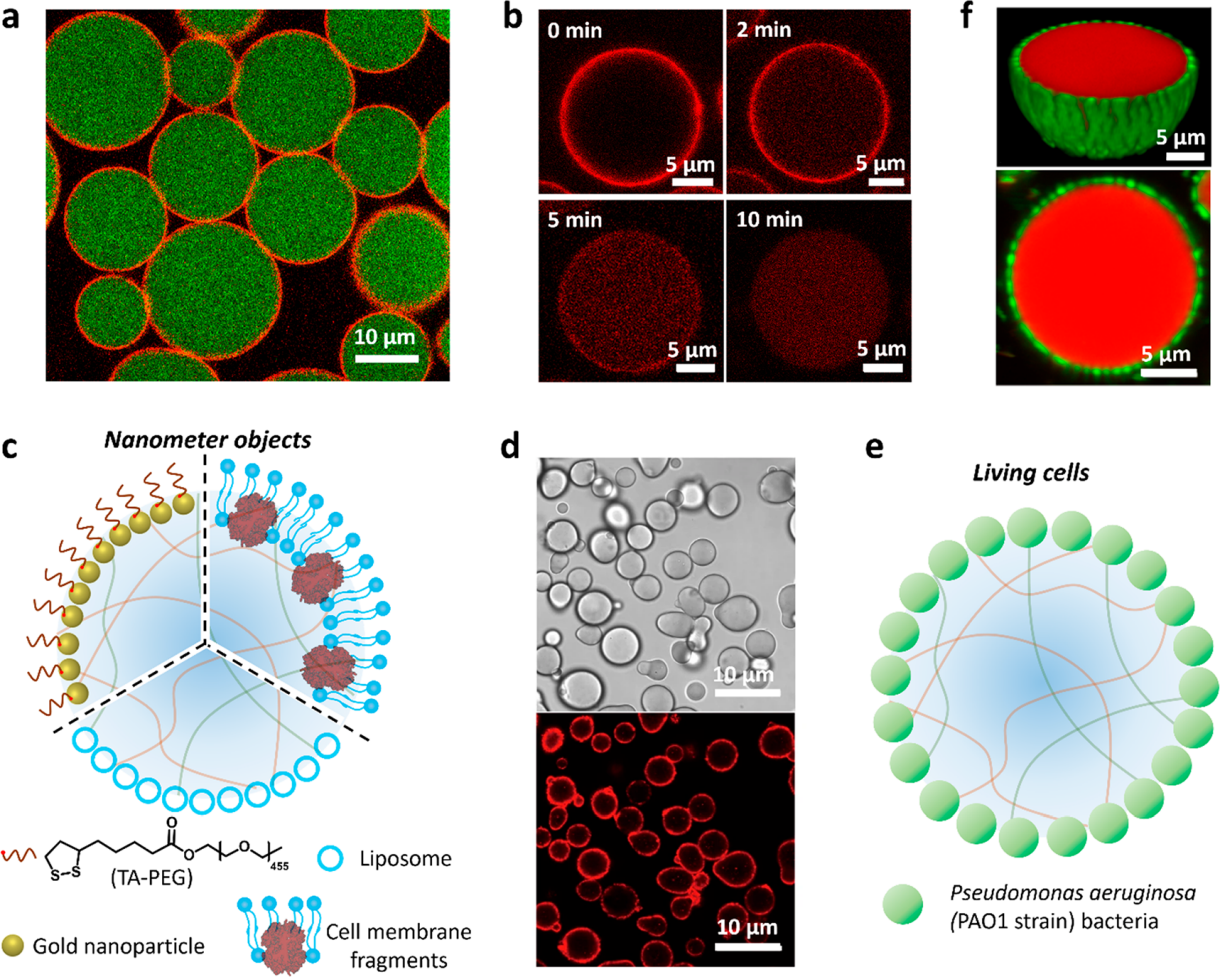
Membranized coacervate microdroplets based on nano- and microscale interfacial self-assembly. (a) CLSM image of caged coacervate droplets; red fluorescence membrane (jammed Au/RITC-labeled TA-PEG nanoparticles); green fluorescence interior (FITC-labeled CM-dextran coacervate).^1^ The Au nanoparticles are initially coated with tannic acid to prevent aggregation. (b) Time series of CLSM images showing changes in membrane and interior red fluorescence for a Au/RITC-TA-PEG nanoparticle-caged coacervate droplet after light illumination for different time intervals. Membrane disassembly and translocation of the decapped Au nanoparticles into the coacervate interior occurs within 10 min. Reproduced with permission from ref (1). Copyright 2022 American Chemical Society. (c) Scheme showing coating of a coacervate microdroplet with nanometer-sized objects; TA-PEG/gold nanoparticles (top left),^1^ liposomes (bottom),^36−38^ or cell membrane fragments (top right).^2,35^ (d) Bright-field (top) and fluorescence (bottom) images of Dil-stained erythrocyte membrane-fragment-encapsulated DEAE-dextran/DNA coacervate microdroplets. Reproduced with permission from ref (2). Copyright 2020 Springer Nature. (e) Scheme showing continuous shell of living *P. aeruginosa* cells surrounding a coacervate microdroplet. (f) 2D CLSM (bottom) and 3D reconstruction (top) images of bacteria-coated PDDA/ATP coacervate microdroplets (*P. aeruginosa*, green fluorescence; coacervate, red fluorescence). Reproduced with permission from ref (39). Copyright 2022 Springer Nature.

Given that nanoscale objects such as amphiphilic polymer–protein conjugates are effective stabilizers of the coacervate/water interface,^32^ we recently developed a membranization strategy based on the reversible jamming of a monolayer of functional inorganic nanoparticles (Figure 3a).^1^ In general, amphiphilic nanoparticles with appropriate wetting properties can be extensively used to prepare water-in-oil Pickering emulsion droplets that are subsequently cross-linked and transferred into all-water media to produce inorganic-based protocells (colloidosomes).^9^ As the free energy (Δ*G*) required to remove a non-cross-linked spherical nanoparticle from an interface is given by
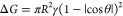
where *R* is the nanoparticle radius, γ the interfacial tension, and θ the contact angle,^34^ the value of Δ*G* associated with the coacervate/water interface is still several orders of magnitude higher than the kinetic energy even though the interfacial tension is extremely low (0.01–0.5 mN/m). Based on these considerations, we recently designed and synthesized new types of nanoparticle surfactants using a combination of a thioctic acid-modified PEG (TA-PEG) polymer and tannic acid-coated gold (Au) nanoparticles as integrated building blocks for the membranization of preformed PDDA/CM-dextran coacervate microdroplets.^1^ As the tannic acid-coated Au nanoparticles and TA-PEG have strong affinities with the coacervate and continuous phases, respectively, *in situ* conjugation of TA-PEG on the tannic acid-Au surface occurs specifically at the coacervate/water droplet interface to generate a closely packed array of Janus-structured surface-active TA-PEG/Au nanoparticles, which cage the coacervate microdroplets (Figure 3a). Significantly, the robust, semipermeable membrane can be partially or completely unjammed by light-mediated dissociation or chemically induced scission of TA-PEG from the Au nanoparticle surface (Figure 3b), endowing the model protocells with programmable release properties.^1^

As an extension to using molecular or nanoscale amphiphiles for coacervate membranization, we replaced these single component building blocks with multicomponent modules comprising embedded functionality (Figure 3c). For example, controlling the balance between hydrophilic and charge–charge interactions results in the spontaneous interfacial assembly of negatively charged hemoglobin-containing erythrocyte membrane fragments on the surface of positively charged DEAE-dextran/dsDNA coacervate microdroplets (Figure 3d).^2^ Similarly, cationic amylose/hyaluronate coacervate microdroplets have been coated in yeast cell fragments.^35^ Other studies have assembled arrays of negatively charged hydrophilic liposomes, ca. 180 nm in size, on the surface of positively charged coacervate droplets to produce model protocells with multicompartmentalized organization (Figure 3c).^36−38^ Finally, in very recent work, we used living bacteria as building blocks for the membranization of PDDA/ATP coacervate microdroplets.^39^ In this system, populations of negatively charged *Pseudomonas aeruginosa* bacteria with diameters of 1–2 μm are trapped specifically at the coacervate/water interface to produce a living cell/coacervate hybrid architecture (Figure 3e,f). Assembly of the membrane into a closely packed array of viable bacterial cells stabilizes the coacervate droplets and facilitates further processing into complex multifunctional bacteriogenic protocells.^39^

## 3 Coacervate Droplet-to-Vesicle Transformations

The above-mentioned strategies employ a range of surface-active components for membrane assembly while maintaining the integrity and homogeneity of the coacervate droplet scaffold. An alternative approach to membranization involves the self-transformation of coacervate droplets into coacervate vesicles by either using auxiliary surface reconstruction agents or employing endogenously driven morphological transitions. In both cases, formation of the membrane-bounded coacervate vesicles occurs in association with an osmotic pressure gradient that is generated by third-party polyelectrolyte complexation at the droplet/water interface (surface reconstruction) or through programmed changes in the charge balance and stoichiometry of the constituent polyelectrolytes (morphological transitions).

**Figure 4:**
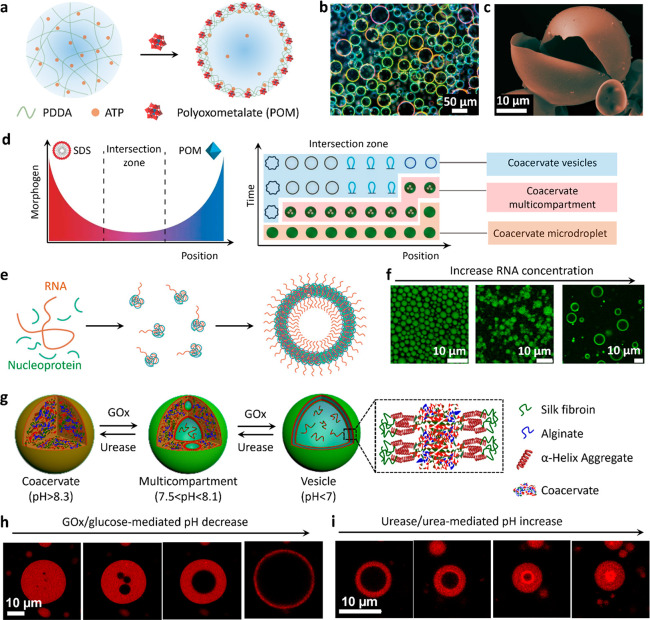
Coacervate droplet-to-vesicle reconfiguration. (a) Scheme showing POM-mediated coacervate droplet-to-vesicle transition via surface complexation and osmotic pressure-induced swelling of PDDA/ATP coacervate droplets.^40,47^ (b,c) Corresponding dark-field microscopy and (c) SEM images of the coacervate vesicles showing the continuous POM/PDDA membrane. Reproduced with permission from ref (47). Copyright 2020 Springer Nature. (d) Scheme showing counter-directional chemical (SDS and POM) gradients and corresponding morphological transitions of PDDA/ATP coacervate droplets within the intersection zone of the different membrane-forming agents. Coacervate vesicles with various structures are produced as a function of relative position and time due to local changes in the SDS/POM ratio. The initial population of identical membrane-free coacervate droplets is transformed into a segregated community of spatially and functionally differentiated protocells. Reproduced with permission from ref (3). Copyright 2019 Springer Nature. (e) Scheme showing coacervate droplet-to-vesicle transition by inducing charge mismatching in coacervate constituents (RNA and nucleoprotein) under substoichiometric (disproportionate) conditions. (f) CLSM images showing the structure of nucleoprotein/RNA condensates at different RNA concentrations. Homogeneous coacervate droplets prepared from stoichiometric mixtures transform into membrane-bounded coacervate vesicles under charge-mismatched conditions. Reproduced with permission from ref (43). Copyright 2020 National Academy of Sciences. (g) Scheme showing enzyme-mediated transitions between isotropic coacervate microdroplets, multicompartmentalized coacervate droplets and coacervate vesicles with a single water-filled lumen and thin outer membrane. The coacervate microdroplets are prepared using CSF and negatively charged alginate.^4^ (h,i) CLSM images showing GOx-mediated alginate/CSF coacervate droplet-to-vesicle transition (h) and reverse transition in the presence of urease (i). Assembly and disassembly of the membrane is associated with an increase or decrease in the osmotic pressure gradient, which in turn gives rise to expansion or contraction of the water-filled lumen, respectively. Reproduced with permission from ref (4). Copyright 2022 Wiley-VCH.

To our surprise, we first observed that homogeneous coacervate droplets are spontaneously transformed into vesicles when a polyanionic polyoxometalate (POM, molecular structure: Na_3_PO_4_·12WO_3_*·x*H_2_O) is added within a minute of preparing a suspension of PDDA/ATP coacervate droplets.^40^ Our original goal was to membranize the coacervate droplets using an interfacial assembly method involving surface complexation of the POM with PDDA at the coacervate/water interface along with retention of the homogeneous coacervate interior. Indeed, addition of the highly charged POM does result in the assembly of a semipermeable polyelectrolyte shell but also induces a drastic reconstruction of the coacervate droplet into coacervate vesicles due to osmotically induced expansion (Figure 4a–c). Consequently, the spherical vesicles consist of a water-filled lumen, a highly compressed submembrane coacervate phase, and a continuous POM/PDDA membrane. Surprisingly, when we undertook these transformations on coacervate droplets organized on glass substrates by acoustic trapping we observed both spherical and elongated forms of the coacervate vesicles depending on the concentration of added POM.^3^ As coacervate droplet-to-vesicle transformations are also induced in the presence of sodium dodecyl sulfate (SDS), we used opposing chemical diffusion gradients of POM and SDS to generate arrays of membranized coacervate microdroplets with spatiotemporally differentiated structure, morphology, and function (Figure 4d).^3^

Based on observations derived from living cells^41^ and RNA-protein complexes,^42,43^ which indicate that coacervate droplet-to-vesicle self-transformations can occur via a process of vacuolization under nonstoichiometric conditions (Figure 4e,f), we designed an endogenous process of self-membranization in coacervate microdroplets prepared from negatively charged sodium alginate and cationized silk fibroin (CSF).^4^ We observed that the homogeneous microdroplets undergo a reversible reconfiguration into coacervate vesicles at excess concentrations of alginate or CSF, which are produced *in situ* by changing the pH using coencapsulated GOx and urease (Figure 4g). As CSF is an amphiphilic polymer consisting of hydrophilic random-coil motifs and hydrophobic α-helical regions, increasing the charge mismatch in the coacervate droplets by GOx-mediated lowering of the pH gives rise to segregation of CSF molecules at the droplet/water interface to produce a semipermeable outer membrane (Figure 4g). Consequently, the increase in osmotic pressure due to membrane formation drives the influx of water, resulting in the growth of a water-filled vacuole, expansion of the droplet and compression of the coacervate phase against the membrane (Figure 4h). The reverse transition is achieved by switching on urease activity so that the pH is increased and charge-matched conditions restored (Figure 4i).^4^

## 4 Cytomimetic Properties of Membrane-Enclosed Coacervate Microdroplets

Cytomimetic properties associated with coacervate membranization include the stabilization of molecular-rich microcompartments against coalescence, customizable permeability that enables selective access to and sequestration of external agents, release of internalized components via triggerable membrane disassembly, and enhanced processing of microscale reaction environments. In this section, we outline some of our recent work and that of others on using membranized coacervates as model protocells.

### 4.1 Selective Membrane Permeability

Unlike lipid vesicles, the permeability of membranized coacervate protocells is determined by the synergism between the molecularly crowded interior and enclosing outer shell. Thus, a client molecule will not gain access to the protocell even when the molecule is smaller than the mesh size of the membrane if the partition coefficient of the solute in the coacervate interior is low.^15^

In general, the semipermeability of model protocells associated with membranized coacervate microdroplets is highly dependent on the fidelity of interfacial assembly. For example, anionic multilayer fatty acid-coated coacervate protocells exclude only negative-charged small molecules due to charge repulsion,^24^ while defect-free phospholipid bilayer-coated coacervate-based protocells are generally impermeable to small molecules.^29^ In contrast, assembly of a disordered lipid bilayer on the surface of DEAE-dextran/DNA coacervate microdroplets gives rise to increased levels of porosity with a molecular cutoff of ca. 4 kDa.^27^ Increasing the size of the membrane building blocks usually increases the porosity. For example, coacervate microdroplets enclosed within a protein/PEG membrane exhibit a molecular cutoff of 10 kDa,^32^ while the membrane produced by the self-transformation of alginate/SCF droplets is highly permeable to solutes below ca. 40 kDa.^4^ Similarly, supported membranes prepared from Au/PEG nanoparticle surfactants are highly porous, with proteins such as GOx (Mw 160 kDa) readily entering the coacervate interior via diffusive transfer.^1^ Interestingly, coacervate droplets coated with a terpolymer-based membrane are permeable to dextran with a molecular weight of ca. 70 kDa despite the molecular size being significantly larger than the membrane mesh.^31^ This was attributed to a combination of flexible membrane dynamics and long-range attractive interactions between the guest molecules and coacervate interior.

### 4.2 Microscale Reactivity and Chemical Signaling

**Figure 5:**
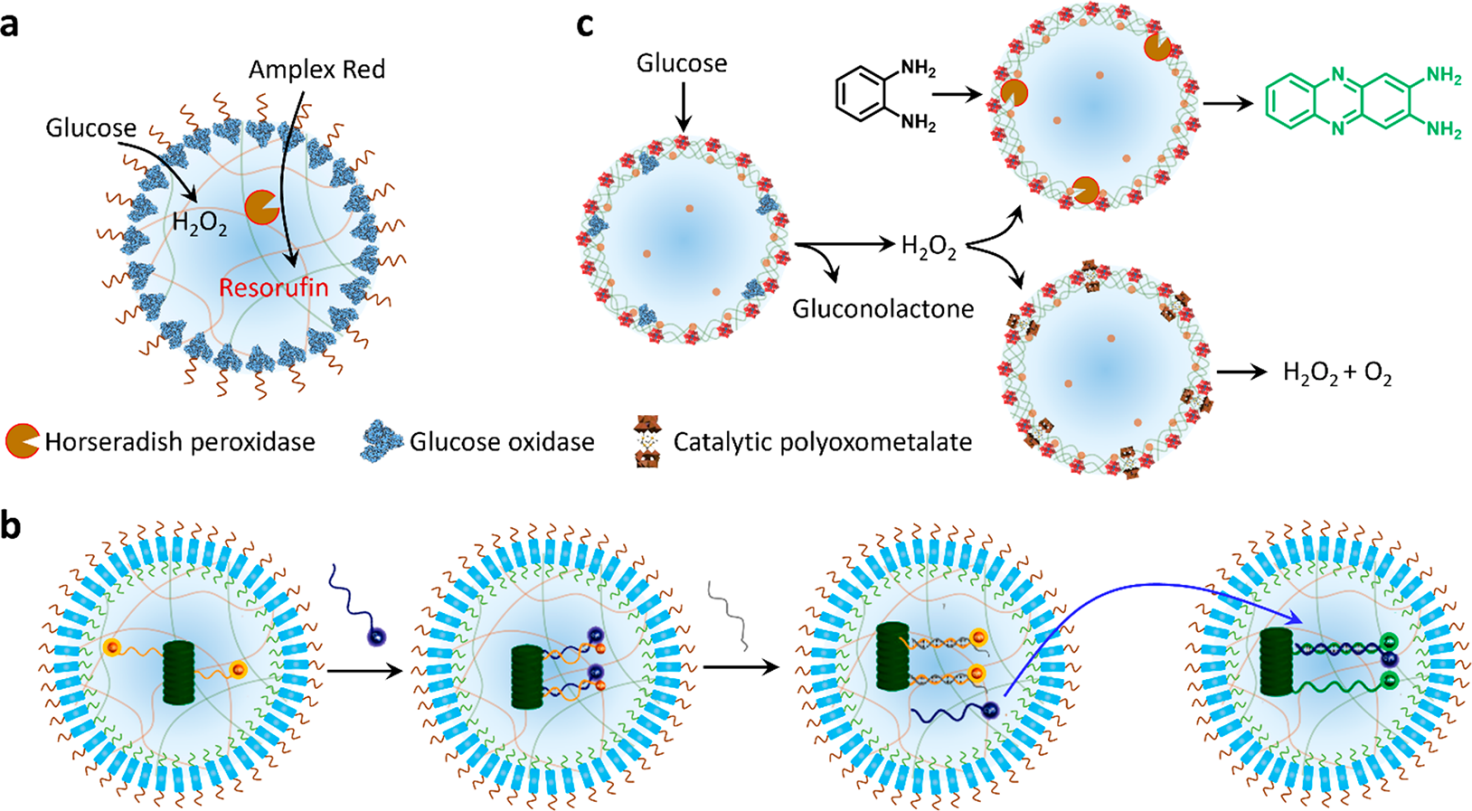
Microscale reactivity and chemical signaling. (a) Scheme showing membrane-to-interior chemical signaling in membranized coacervate droplets by integration of a PEG-modified GOx membrane and HRP-containing coacervate interior.^32^ (b) Scheme showing DNA signaling in populations of terpolymer (turquoise) membrane-enclosed semipermeable coacervate droplets containing a DNA-decorated nanoscaffold (dark green). A complementary ssDNA input strand (blue) acts as a fluorescence reporter that can be displaced by a fuel strand (gray) (left side). Efflux of the reporter strand is used as a communication signal (blue arrow) for activating a second population of the protocells (right side). Reproduced with permission from ref (46). Copyright 2020 American Chemical Society. (c) Scheme showing chemical communication pathways in a community of coacervate vesicle-based protocells consisting of GOx/POM, HRP/POM, or RuPOM catalytic membranes. Hydrogen peroxide is generated by the GOx/POM population and employed as a diffusive signaling molecule for competing transformations in the HRP/POM (peroxidase activity) and RuPOM (catalase-like activity) populations. Reproduced with permission from ref (47). Copyright 2020 Springer Nature.

Membranization of coacervate microdroplets provides an effective pathway to the fabrication of reactive protocell-based microcrucibles that engage in internal or external chemical communication. In particular, the interfacial assembly strategy enables modular functions on the membrane and coacervate milieu to be integrated, providing a potential toolbox for implementing catalytic reactions and establishing embodied chemical networks. For example, GOx-bound coacervate microdroplets with HRP-enriched interiors have been used to perform a spatially organized enzyme cascade in which chemical signals (hydrogen peroxide, H_2_O_2_) are sent from the membrane surface and received within the molecularly crowded core (Figure 5a).^32^ Other studies have used membranized coacervate microdroplets for chemical-mediated information exchange either within single protocells consisting of subcompartments^44^ or among different protocell populations.^45^ Alternatively, terpolymer-coated coacervate droplets containing a supramolecular nanoscaffold have been used to generate programmable communication networks based on DNA strand displacement reactions (Figure 5b).^46^

**Figure 6:**
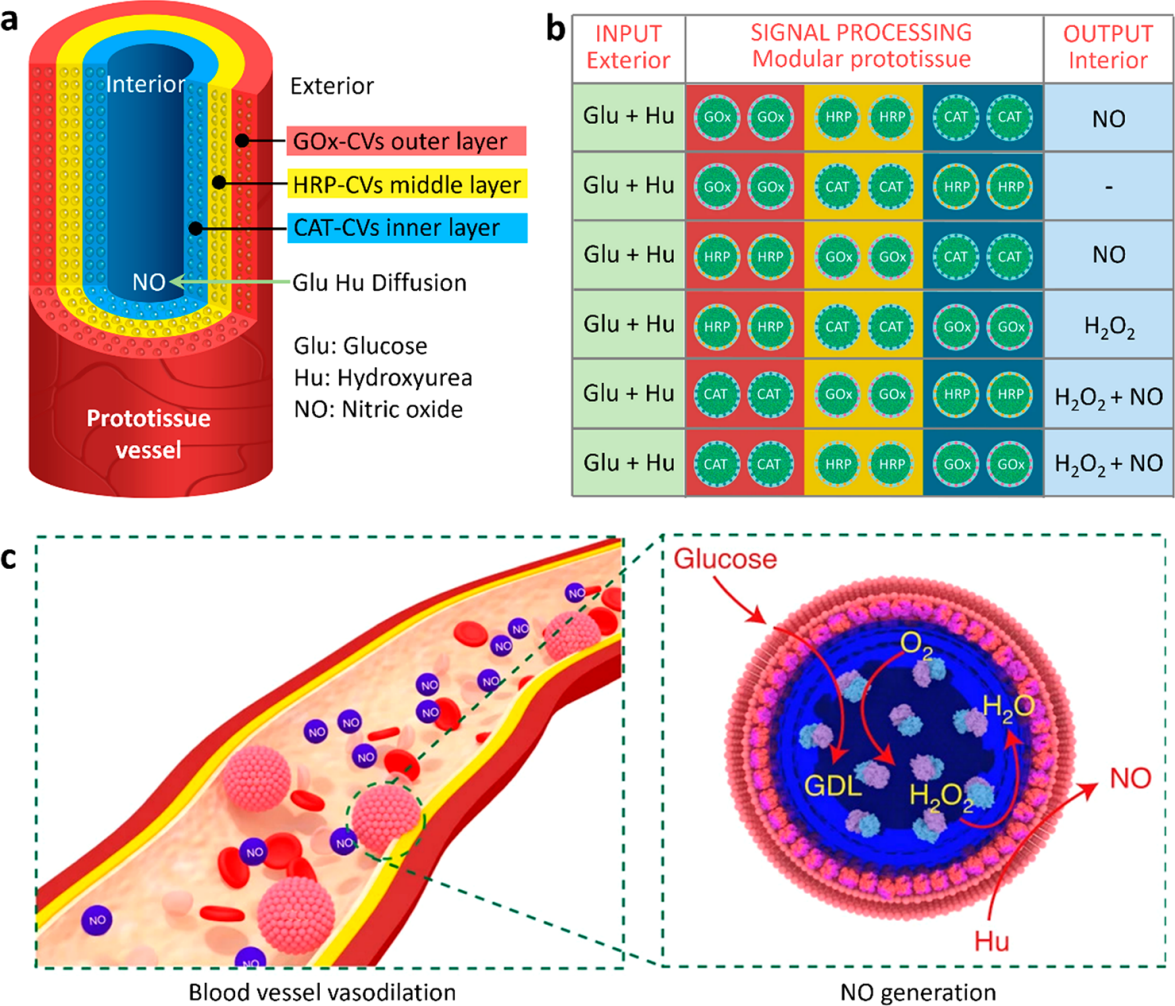
Prototissue construction and therapeutic protocells. (a) Scheme showing three-layer prototissue-like vessel harboring phospholipid-enveloped PDDA/DNA coacervate vesicles (CVs) decorated with GOx in the outer hydrogel layer, HRP in the middle hydrogel layer and catalase (CAT) in the inner hydrogel layer.^28^ (b) Varying the spatial sequence of the enzyme-CV modules in the presence of identical exterior inputs (Glu and Hu) gives rise to different outputs in the internal lumen. Reproduced with permission from ref (28). Copyright 2022 Springer Nature (c) Scheme showing the design of membranized coacervate microdroplet-based therapeutic protocells that perform GOx/hemoglobin-mediated generation of NO in the presence of glucose and hydroxyurea (Hu) for blood vessel vasodilation. GOx and hemoglobin are spatially positioned in the coacervate interior and membrane, respectively. Reproduced with permission from ref (2). Copyright 2020 Springer Nature.

In our work, we used a surface reconstruction pathway to prepare PDDA/ATP coacervate vesicles with a catalytic ruthenium-polyoxometalate (RuPOM) membrane that facilitates catalase-like H_2_O_2_ decomposition and O_2_ production.^47^ By incorporating competitive H_2_O_2_ decomposition (RuPOM) or peroxidase (HRP) reaction pathways within individual protocells, spatially distributed signaling pathways capable of parallel catalytic processing are established. Specifically, we encapsulate GOx in a population of the POM-enclosed protocells and add glucose to send a H_2_O_2_ signal to two other populations comprising HRP-loaded POM-bounded coacervate droplets or RuPOM coacervate vesicles (Figure 5c). Competing transformations associated with the HRP/POM and unloaded RuPOM protocell populations then give rise to parallel processing of the diffusive signal. In other studies, we developed an enzyme-decorated membranized coacervate droplet integrated system with dual-substrate inputs that result in logic-gate signal processing under reaction-diffusion conditions.^28^ To achieve this, we prepared phospholipid-enveloped PDDA/DNA coacervate droplets decorated with GOx, HRP or catalase and immobilize each single population in three separate hydrogel modules to construct a tubular prototissue-like vessel capable of modulating the output of NO (Figure 6a). Arranging the modules concentrically into a three-layer tube and inputting glucose and hydroxyurea from the external environment results in distinct NO outputs in the internal lumen depending on the spatial organization of the three processing domains (Figure 6b). Significantly, the NO output reduces the level of platelet activation and blood clot formation in samples of plasma and whole blood located in the internal channel of the device, suggesting that the device could be developed for anticoagulation applications.^28^

In related work, we prepared aqueous suspensions of GOx-containing coacervate droplets enclosed within hemoglobin-containing erythrocyte membrane fragments (Figure 3c,d) and explored the potential use of the protocells as *in vitro* and *in vivo* NO-mediated vasodilation agents (Figure 6c).^2^ The coencapsulated enzymes generate a flux of NO in the presence of both hydroxyurea and glucose, and synergistic incorporation of the cell membrane fragments and enzyme-loaded coacervate interior significantly increases the biocirculation times and hemocompatibility, offering potential advantages for the use of membranized coacervate microdroplets in biomedicine, cellular diagnostics and biomedical engineering.^2^

### 4.3 Artificial Phagocytosis

**Figure 7:**
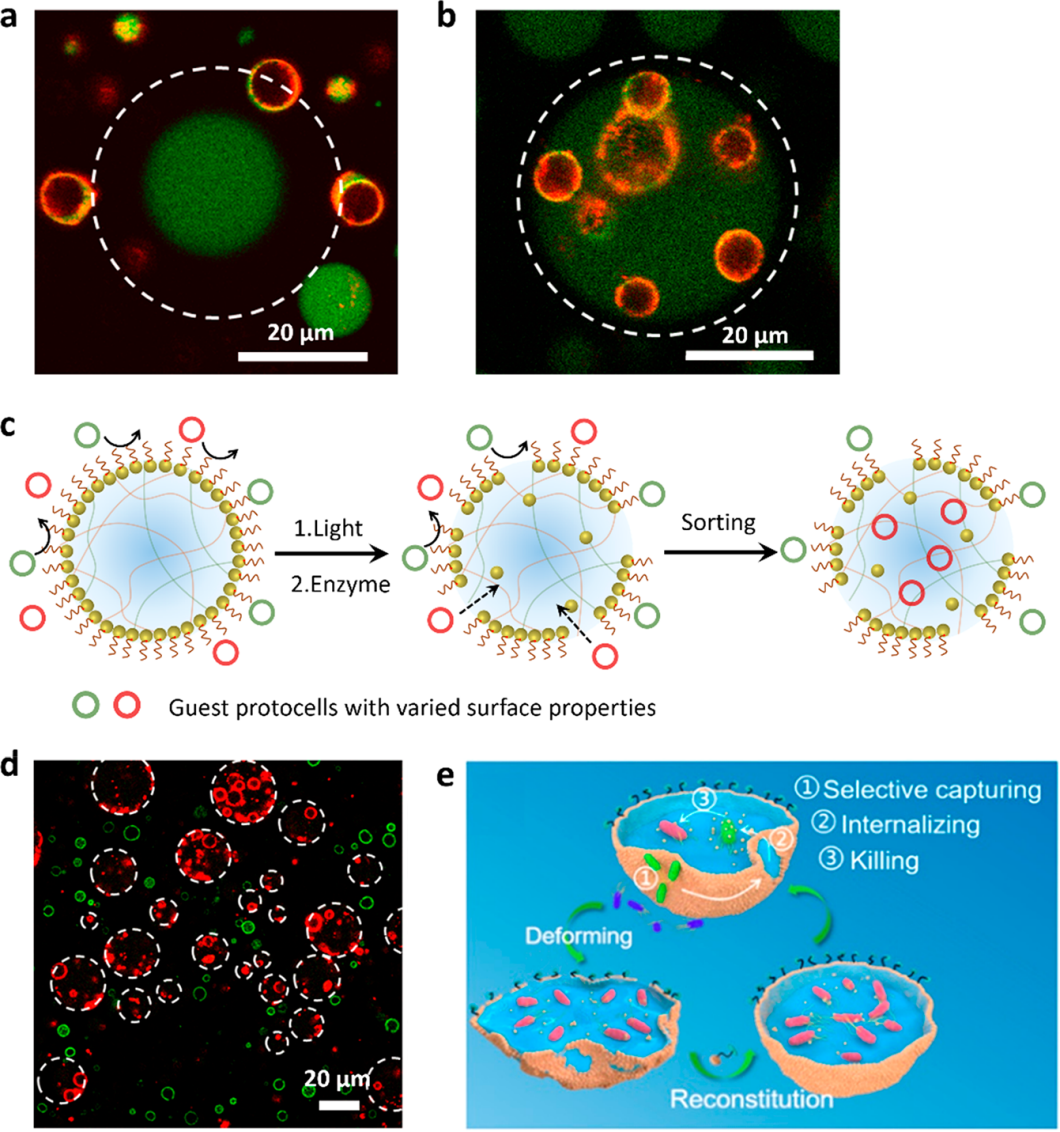
Artificial phagocytosis and protocell sorting. (a,b) CLSM images of red/green fluorescence overlay images showing a single FITC-labeled GOx-containing Au/TA-AE-PEG6k-caged coacervate droplet surrounded by RITC-labeled PCVs and recorded before (a) and 60 min after (b) addition of glucose. Unjamming of the membrane by enzyme-mediated cleavage of polymer TA-AE-PEG6k results in PCV transfer across the membrane. The focal plane is aligned with the PCVs not the caged coacervate droplet. White dash circles delineate the boundary of the caged coacervate droplet.^1^ (c) Scheme showing triggered uptake and sorting of guest protocells in Au/PEG nanoparticle-caged PDDA/CM-dextran coacervate protocells via the light or enzyme-mediated disassembly of the membrane.^1^ (d) CLSM images showing the selective capture of RITC-labeled PCVs (red fluorescence) within the interior of the nanoparticle-caged protocells (white dashed circles), while FITC-labeled PEG-modified colloidosomes (green) remain in the external environment. Reproduced with permission from ref (1). Copyright 2022 American Chemical Society. (e) Scheme showing artificial phagocytosis of *E. coli* bacterial cells within membranized coacervate microdroplets. The membrane is generated by the interfacial assembly of yeast membrane fragments and is locally disrupted by mechanical agitation, leading to aperture formation and capture of the bacteria, which subsequently die in the presence of cationized amylose. Addition of yeast membrane fragments results in resealing of the apertures. Reproduced with permission from ref (35). Copyright 2021 American Chemical Society.

Recent studies from our laboratory demonstrate that coacervate-based microdroplets spontaneously ingest colloidal objects such as enzyme-loaded proteinosomes to produce host–guest protocells capable of synergistic and antagonistic chemical coupling.^48^ While capturing external microscale objects through a membraneless interface seems readily achievable in general, the design of similar behavior in membranized coacervate droplets is likely to be considerably more difficult. As a first step toward this goal, we prepared a mixed population of Au nanoparticle-caged coacervate microdroplets and POM-membranized coacervate vesicles (PCVs) as described above (Figures 3a and 4a, respectively) and triggered microscale aperture formation in the Au nanoparticle membrane using light or enzyme-mediated changes in pH. Consequently, the smaller POM-enclosed protocells transfer into the interior of the larger Au nanoparticle-stabilized coacervate droplets in a process of artificial phagocytosis (Figure 7a,b). Interestingly, because the contact-induced capture of the guest protocells derives from the high sequestration potential of the coacervate core once exposed in the host protocell, tailoring the surface chemistry of the client protocells, for example, by using both POM-membranized coacervate vesicles and PEG-grafted colloidosomes, provides a rudimentary system of protocell sorting (Figure 7c,d).^1^ Others have used mechanical agitation to generate apertures in yeast cell fragment-coated amylose/hyaluronate for the selective capture and predation of living bacteria (Figure 7e).^35^

## 5 Conclusions and Prospects

This Account highlights recent advances in the design and construction of membranized coacervate microdroplets and their applications as protocell models with cytomimetic properties. By highlighting our own work and that of others, we present two different approaches to membrane assembly: (i) interfacial assembly using surface-active building blocks with a wide range of length scales from the molecular to microscopic; and (ii) coacervate droplet-to-vesicle reconfiguration via interfacial interactions with auxiliary surface reconstruction agents or by endogenously triggering structural and morphological transitions under nonstoichiometric (charge mis-matched) conditions. In general, interfacial assembly give rises to membranized molecularly crowded model protocells with a well-delineated ultrathin outer membrane of added building blocks that enclose a homogeneous coacervate interior. In contrast, surface reconstruction leads to a more diffuse coacervate-based membrane of variable thickness along with an aqueous lumen. In both cases, we show that membranization results in a range of cytomimetic properties, including selective membrane permeability, microscale reactivity and chemical signaling, and artificial phagocytosis, which provide opportunities for developing new technologies at the synthetic cell/living cell interface.^49^

Given the relative simplicity of preparing coacervate droplets by associative liquid–liquid phase separation, it should be possible to develop the methodologies described in this Account as a systematic approach to a wide range of molecularly crowded membrane-bound protocell models with different compositions, sizes, internal organization, and properties. In our studies, membranized coacervate microdroplets with diameters ranging from a few micrometres to tens of micrometers could be readily constructed by controlling droplet nucleation, growth, and coalescence through fine-tuning the polyelectrolyte concentration, charge ratios, and time intervals prior to the onset of membrane assembly. However, in each case, only spherical cell-like objects are produced; therefore, it remains a challenge to develop methodologies for preparing membranized coacervate microdroplets with controllable shape anisotropy and asymmetric organization.

Although much progress has been made in recent years, there remains some key limitations that will need to be addressed in future work. First, the selective uptake of small molecules is difficult to achieve for most systems currently developed. Although it may be possible to augment defect-free phospholipid-based membranes with molecular porins for use in giant coacervate vesicles, the general onset of structural disorganization during membrane assembly at the coacervate droplet/water interface requires the development of postconstruction annealing or resealing strategies. Second, reversible electrostatic interactions between the coacervate constituents as well as at the interface with the membrane building blocks render the protocell models sensitive to excessive dilution and high ionic strengths. Developing robust counterparts is a prerequisite for many potential applications and approaches based on postassembly covalent modifications may be required. Third, the general use of highly charged cationic polymers for coacervate droplet assembly may be a problem for therapeutic applications involving living cell/synthetic cell interactions. A possible solution is to cloak the protocells with a biocompatible shell, as demonstrated by recent studies in which hemoglobin-containing erythrocyte membrane fragments were assembled on the surface of DEAE-dextran/dsDNA coacervate microdroplets to improve hemocompatibility and increase blood circulation times.^2^
